# Treatment of Fractures and Contusions of Articulations, as Applied to the Elbow-Joint

**Published:** 1872-07

**Authors:** W. F. Peck

**Affiliations:** Davenport, Iowa, Professor of Surgery, Medical Department Iowa State University


### TREATMENT OF FRACTURES AND CONTUSIONS OF ARTICULATIONS, AS APPLIED TO THE ELBOW-JOINT.

BY W. F. PECK, M.D., DAVENPORT, IOWA,
*Professor of Surgery, Medical Department Iowa State University.*

[Read Before the Iowa State Medical Society, June 27th, 1872.]

Scarcely any special class of injuries is so unsatisfactorily treated as those which occur in and around articulations. The cause of the manifest suppression of opinion regarding results of treatment in this numerous class of cases is due to *two facts:* First—The termination of the case, after having received the best treatment recommended by the books, usually exhibits more or less deformity. Second—To acknowledge that the result is not good or satisfactory, would be to supply evidence that might encourage suits for *malpractice*, inasmuch as deformity is a frequent result of *contusion*, *extra* or *intra-articular*. The cause should be critically examined, and if a remedy exists in our art, it should be advocated and adopted.

Having been myself the subject of severe censure at the hands of those who were unskilled, practically, in the management of injuries of joints, for, as alleged, unwarrantable treatment—although the result was perfection approximated—I propose considering, briefiy, the cause and the remedy.

Entering into the formation of all movable joints, there exists, anatomically, bone cartilage, synovial membrane, synovial fluid, ligamentous tissue—which is almost absolutely inextensile—connective and cellular tissue, including the nervous and vascular distributions. The *lymphatic* tissue is unimportant in connection with the subject under consideration. *Force*, when applied, may only contuse or injure the soft parts, not including the ligaments (or hinges) connecting bone. In any case, if the injury be of any magnitude, blood-vessels are lacerated, and a solution of continuity takes place in a few or many of the nerves, as the case may be. The result of the injury to the vessels is that their contents are permitted to ooze either slowly or rapidly around, partially or completely, the articulation. The presence of the effused fluid upon injured and normal tissue contributes, in connection with solution of continuity in the nerve-tissue, to cause pain. The pain and swelling *seem* to indicate absolute *rest* so long as they exist. The fluid thus abnormally located, elementally contains water, *fibrin,* iron, albumen, and a few salts—all of which are reappropriated, especially the fibrin, with more or less difficulty. The treatment almost universally advised for such a pathological condition is immobility of the parts (the joint), and to secure that end, splints are resorted to. Sometimes contusion is not restricted exclusively to the soft parts, particularly to the elbow-joint—and it is to *this joint* that I desire my views to more especially apply—but complicates both ligamentous and osseous structures. When the tissues last named are involved, the swelling from effusion and secondary changes is greater than when the soft parts are alone the seat of unnatural results, and the pain is correspondingly increased. If it is the correct treatment to confine absolutely the parts around the joint in cases of contusion, the reasons for absolutely confining the joint when some of its component parts are dissolved, are still greater! In the treatment of fractures, *rest,* as absolute as may be, is encouraged, that union of the uncontinuous bone may be effected. It is safe to state, that if a surgeon should depart from the above general assumed indication in the treatment of special cases, unless he could satisfactorily account for a departure, he would be unsupported in defending himself against alleged *malpractice,* by the practical treatment of to-day.

In the application of dressings in the treatment of fractures, one *special* indication, after the bones have been reduced, is to be observed as completely as possible, namely: *fixed points* are made upon one or more places upon the separated segments. Unless they can be made, no guarantee exists that deformity will not result. Even when they can be obtained, there are instances when, to make them and continue them uninterruptedly, deformity—that of itself may furnish ample grounds for a suit for malpractice—may result. In fractures of the humeral condyles complicating the joint, I feel authorized, from practical and observational experience, to state that two fixed points, after the bones have been completely reduced—a result which I do not believe possible—one on the separated fragment, and the other on the main bone *can not be accomplished.* In case the separated bones have been approximated, in order to secure union, the indication heretofore mentioned—*absolute rest*—is to be made, and that is almost universally done with splints of one kind or another. If the splints are kept applied to the parts, deformity, to a greater or less extent, will almost always occur; and when it does occur, if it chance to be moderate, the result will be considered a good one. This decision, with reference to *elbow-joints* especially, is not correct, when the correct treatment, based upon practical results, is regarded. In the first place, it is not possible—with due respect to those who have furnished text-books which advocate opposite opinions—to reduce the humero-condyloid fragments completely, and maintain them, by dressings, in position. If such a result were possible, it would be almost impossible to obtain a result without greater or less anchylosis. The requisite amount of time necessary to secure *union*, everything being equal, is at least from *four* to *six* weeks. If a joint without injury should be kept absolutely quiet for the same length of time, stiffness, to a considerable degree, would exist. To the splint quietude of a joint, add the pathological changes which take place when an effusion with inflammatory changes occurs, and you have, as a necessary result, a want of natural mobility of the joint resulting.

This leads us to inquire if a treatment can be supplied which will furnish more desirable results. About a quarter of a century since, Dr. J. C. Warren, of Boston, seemed to recognize the great difficulty in the way of obtaining non-anchylosed joints after contusion and fracture, and he advocated, as a preventive means, movement of the joint occasionally after the injury. Few surgeons have sufficient independence of action to permit them to deviate from the rules and practice of their predecessors, whether the results of the operation of those rules and practice are all that could be desired or not.

Frequently, after an injury of the elbow, we are unable to determine whether the contusion is confined to the soft tissue, or whether the force operating has gone further, and produced a solution of continuity in the osseous structures; in any event, the chief object advised in the application of treatment is to prevent deformity. And how is it to be best avoided? Are splints and dressings to be employed with the view of keeping the parts absolutely at rest? If used at all, for what purpose are they to be used? Splints, of whatever kind, are to be considered only supportive, not retentive. They should be removed each day—or each alternate day may suffice—and movement of the joint should be made. Many will undoubtedly, at first thought, censure this recommendation, but considerate judgment can not do otherwise than approve of the results obtained by this seemingly unwarrantable practice. Why move the joint and disturb the fracture? Because the absolute rest encouraged in order to cause union, and the union which is always imperfect as to direct relation, produce anchylosis. The results of moving the joint are infinitely to be preferred over those which occur from the stationary plan of management. It may seem, to those who have not tried it, that much pain is caused; and further, that the danger from inflammation might be very great—in fact, so much so that the deformity is preferable to the possible result of such treatment. When either of the condyles are fractured, the degree of displacement is considerable; it may be laterally below, above, or between any of the cardinal points named. If it is downward or sideways, the tendency is to unnaturally limit the capacity of the joint. Such misplacement, in addition to the effusion, may easily produce partial or complete obliteration of the articulation, and especially if the parts are kept confined immovably with splints. It is far better, as a possible result of movable treatment, to encounter non-union of a condyle than to meet with anchylosis. It is well-established fact, that pressure will promote absorption. Now, if the parts are carefully taken from the supportive splints, and passively moved, the pressure thus made will induce the absorbents to take up and carry away the fluids which might, if permitted to remain uninterfered with, take on semi-organization, and thereby diminish the capacity of the joint.

Again: if the parts are moved as often as the particular case suggests, it is impossible for union of bone or other matter to organize—to take place in such a way as to prevent a useful joint.

While I am fully satisfied that the views herein advocated will ultimately prevail, I feel like cautioning all, who may possess the necessary courage to try the movement plan, against too much and too reckless motion—for I have no doubt that harm may be done by those who are inconsiderate in applying the means.

The following cases are briefly related to illustrate the treatment advocated:

I.—J. G., æt. seven years; native of Iowa; residence, Soldier's Orphan's Home, Davenport, Iowa. In the Spring of 1865, while playing with some playmates, he was thrown to the right side; and in falling, he extended his arm to save himself, but received the force in such a way as to contuse the elbow and fracture the internal humeral condyle. He was treated in Hospital by having the elbow placed upon a pillow, with no splints, and cooling lotions applied to swollen parts. The arm was fixed firmly each day, and the forearm moved carefully, so as to prevent adhesive formations from restricting the natural movements of the joint. When he was permitted to get up and walk about, splints were applied for supportive purposes. The treatment was continued as commenced, until it seemed to be no longer indicated; and at this writing, no difference is to be discovered in the movements of either elbow. Upon close examination, the condyle is moved upward and inward, but it is firmly united by *bony* tissue.

II.—Bessie P., aged four years; native of Davenport, Iowa. While standing in a folding army-chair, with a carpet back, one side slipped off the post, and she was thrown backward, with the right arm twisted under. The fall fractured the internal condyle; subsequently there was considerable swelling. I placed the arm in splints, and each day removed the dressings, and made passive motion. She now has almost complete use of the elbow, and, in fact, no difference can be recognized to exist between the arm that was injured and the unharmed one.

III.—Annie S., aged eight years; native of Davenport, Iowa. Was playing with some companions, when she accidentally slipped, and received the weight of the body upon the right elbow, fracturing the external condyle, and producing therewith considerable contusion of the adjacent soft parts. The arm and forearm were supported by a binder's board rectangular splint, which was retained in position by a loosely applied bandage. The arm was removed from the dressings each day, and a sufficient amount of motion made to assist in causing absorption, and to prevent union of bone taking place at an angle that might limit the natural capacity and freedom of the joint. At the end of the first week, the patient was directed (and she complied with the instructions faithfully) to make passive motion of the forearm with the splint applied. At the end of four weeks, union without much displacement having taken place, the dressings were permanently removed, and the motion continued. Six months after the accident the freedom of movement was quite satisfactory; indeed, it required a hypercritical vision to locate the joint in which the previous injury occurred.

I have stated the above cases out of a collection of some fifteen treated by the movement plan, because they were typical cases of fracture and contusion combined. The majority of medical men, who treat surgical cases, believe in and employ topical application to reduce swelling. Although it may seem inconsistent, I announce the fact as a result of experience, that the tumefaction which occurs will subside as quick, if not *more* rapidly, by moving the parts, than when absolute quiet is maintained, and when antiphlogistics are vigorously applied.

In accepting the views of Conheim, Recklinghausen, and Simon, upon inflammation, the value, or rather the explanation made as a cause of the beneficial results of the treatment recommended, is seen in the pathological operations of the *leucocyte*, which undeniably possesses an independent life, and which plays an important part both in reparative and destructive inflammation. From the fact that the white corpuscle, or the *leucocyte*, is not unlike, microscopically, the pus-globules, and that it exists naturally in the vital current—that it possesses the ability to insinuate itself between the cell structures of the capillaries—and recognizing the fact that it can and does thus migrate, it can readily be understood how it can be persuaded to return to its original avenue of operation. Not unfrequently, improperly treated inflammation terminates in suppuration, and, as a secondary consequence, compound fracture occurs. A want of vitality, encouraged by a condition of stasis, will cause either anchylosis, more or less completely, or suppuration; whichever condition happens, permanent injury of the joint is likely to supervene. When judicious movement of the joint is practiced, in accordance with the indications of the case under observation, stasis, ending in semi-organization of matter or suppurative inflammation, is much less liable to occur. Were I to be called into *court* to testify concerning a case of alleged malpractice, wherein the elbow-joint had been contused and complicated by fracture of any of the articulating surfaces, and when said injury had received the absolute quiet plan of treatment, I could not do otherwise than give the plaintiff the benefit of testimony rendered against the defense.

